# Supplementary material for: A non-lethal stable isotope analysis of valued freshwater predatory fish using blood and fin tissues as alternatives to muscle tissue
Source: PLoS One. 2024 Jan 18;19(1):e0297070. doi: 10.1371/journal.pone.0297070 (PMC10796030; doi:10.1371/journal.pone.0297070)
Supplement: Attachment — Submitted filename: PLOS One Vejrik et al 2023 Review.docx [file pone.0297070.s002.docx]

PLOS One Vejrik et al 2023 Review

“A non-lethal stable isotope analysis of valued freshwater predatory fish using blood and fin tissues as alternatives to muscle tissue”

Abstract:

- Can you say that the blood and fin clips are less invasive to the muscle biopsy if none of the approaches were lethal to the fish? Are there studies you can cite in the introduction or discussion that examined fish response and performance after each sampling technique was used to support this claim of “less invasive”?
- What do you mean by “blood proved to be more accurate alternative”? In what sense is it more accurate – does it correspond better to the muscle? Change “more accurate” to “provided better correspondence to muscle isotope values”
- How can blood and muscle “be used interchangeably” but they also “vary significantly” as seen in the ANOVAs?

Introduction:

- Remove period after “Approximately” in first paragraph.
- More elaboration on why blood sampling is preferable to non-lethal muscle biopsy would be useful in paragraph 2.
- Reorder citations in paragraph 2 [31,17] 🡪[17, 31]

Methods:

- “samples of tree body tissues were collected” should be “three”
- Include your alpha value in data analysis
- Need to include what diagnostics you performed for the linear regression analysis & please state whether you examined your residuals

Results:

- Remove “statistically” from all “statistically significant”, it is redundant
- Report SE instead of SD in table 2
- Would not say “The blood generally showed only slight enrichment in D13C compared to the muscle tissue” given that it was 50/50 enrichment/depletion in table 2.
- Edit: “The degree of these differences **was** more pronounced in the blood compared with fins of European catfish**”** In this sentence what do you mean by “it was lower for Northern Pike”? The degree of differences between blood and fin look similar in the two species.
- Last sentence of paragraph 2 calls the blood and fin samples “non-lethal” tissue, but the muscle sampling did not kill the fish either and would therefore also be considered “non-lethal”. Throughout the text, the blood and fish samples are called “non lethal” which is an unfair distinction from the biopsy that was also non lethal.
- First sentence in paragraph 3: remove “the” in “the both D13C and D15N offsets”
- Paragraph after table 3: “however the difference between fin and muscle was statistically insignificant” 🡪 “however no difference was found between fin and muscle”
- Change any phrases of “difference was insignificant” to “no difference was found” or “there was no difference”
- Consider using a linear model with the random effect of site instead of separate regressions for each site (if you are interested in calculating an overall conversion equation for each fish and tissue type). If you want a conversion equation for each lake then fixed effect of site.
- Last paragraph of results rephrase first two sentences: “No distinct trends were found through linear regression analysis between isotopic tissue offsets and fish body mass in either species or in any study site.”
- I recommend again running a random effect linear model on the tissue offsets and body mass (random effect of site) and reporting the test statistics from those model runs rather than averaging R squared values from multiple different linear regressions.
- Need to report the results of model regression diagnostics.

Discussion:

- From my understanding it seems like the muscle biopsy is not lethal to moderate-large sized predatory fish, and so the blood/fin vs muscle argument may be redundant. How does blood sampling affect smaller fish, where taking a muscle biopsy can be particularly invasive/lethal? Does blood sampling impose any negative effects on the fish compared to a biopsy punch? What is the advantage of blood sampling when the biopsy punch is already non-lethal approach to white muscle samples?
- Can you include general turnover rates of each of the tissue types sampled?
- Any inferences on why your fin D15N values were enriched compared to muscle?
- Bold claim to make that “blood and muscle tissues can be used interchangeably in SIA without requiring conversion”, given that you showed that the isotope offsets were significant in 5/8 of your paired t-tests for d13C and 6/8 for d15N, and linear regression models were all significant. This claim is not substantiated.
- “Thus the D13C and D15N values between tissues **may have been in better agreement in these larger adult fish**” instead of “were apparently more stable over time”
- “In conclusion, both d13C and d15N **values** for blood and fin **serve** as highly accurate predictors of **respective isotope values for** muscle tissue **in** adult apex predators…”
- Change “and exhibits a closer resemblance” to “as it corresponds more closely”
- Remove “significant” in “were notably significant different”
